# Pre-operative serum VCAM-1 as a biomarker of atrial fibrillation after coronary artery bypass grafting

**DOI:** 10.1186/s13019-017-0632-2

**Published:** 2017-08-18

**Authors:** Leanne Harling, Jonathan Lambert, Hutan Ashrafian, Ara Darzi, Nigel J. Gooderham, Thanos Athanasiou

**Affiliations:** 1 Department of Surgery and Cancer, Imperial College London, London, UK; 2 Department of Cardiothoracic Surgery, Hammersmith Hospital, London, UK; 3 Department of Biomolecular Medicine, Imperial College London, London, UK; 4 Institute for Child Health, University College London, London, UK

**Keywords:** Post operative atrial fibrillation, Surgery, Vascular cell adhesion molecule, VCAM-1, Biomarker

## Abstract

**Objective:**

Systemic inflammation is a recognised contributory factor in the pathogenesis of de novo post-operative atrial fibrillation after cardiac surgery. This study aims to determine whether serum soluble vascular endothelial cell adhesion molecule (sVCAM-1) may predict the onset of POAF in patients under going coronary artery bypass grafting.

**Methods:**

34 patients undergoing non-emergent, on-pump CABG were prospectively recruited. Plasma was obtained at 24 h pre-operatively and at 48 and 96 h post-operatively. POAF was defined by continuous Holter recording. Inter-group comparisons were performed using student t-test or ANOVA as appropriate.

**Results:**

Thirteen (13/34) patients developed POAF at a mean of 2.5 days post-operatively. Serum sVCAM-1 was significantly increased in the pre-operative serum of POAF when compared to non-POAF patients (*p* = 0.022). No significant difference was observed between the groups at 48 h (*p* = 0.073) or 96 h (*p* = 0.135) post-operatively. sVCAM-1 had a sensitivity of 60.0% and specificity of 77.27%, with an overall diagnostic accuracy of 75.2% in predicting POAF.

**Conclusions:**

sVCAM-1 concentration in the pre-operative serum of patients undergoing CABG may accurately predict the onset of de novo POAF. As such, serum sVCAM-1 may be used as a predictive biomarker for this common arrhythmia. Further work must now perform prospective, targeted validation of these results in a larger patient cohort.

## Background

De novo post-operative atrial fibrillation (POAF) may significantly increase morbidity and mortality after cardiac surgery both in the short- and long-term [1–3]. Furthermore, through increases in hospital stay and resource utilisation it may confer considerable increased costs to patient care [4, 5]. Although factors such as infection and electrolyte imbalance are well recognised to increase the risk of POAF, the pathophysiology of this arrhythmia is multifactorial and involves a complex interaction of ‘triggering’ stimuli and ‘sustaining’ processes acting on a myocardial substrate that may be pre-disposed to developing tachyarrhythmia. It is the exact nature of this atrial substrate that seems to predispose some patients and not others that remains poorly understood.

Vascular cell adhesion molecule 1 (VCAM-1) is an 81 KDa sialoglycoprotein expressed by cytokine activated vascular endothelium, dendritic and macrophage-like cells. It is a member of the immunoglobulin superfamily, which through interaction with integrins present on eosinophils, basophils, mast cells, monocytes and other lymphocytes mediates signal transduction and/or cell adhesion and transendothelial migration [6]. VCAM-1 expression is regulated by a number of factors including inflammatory cytokines (TNF-α, IL-1β), and stimulation of toll like receptors on endothelial cells, fibroblasts and dendritic cells. Furthermore, VCAM-1 gene expression is regulated by the transcription factor NF-κB, which may be activated by TNF-α and reactive oxygen species. Release of soluble VCAM-1 (sVCAM-1) into the circulation has not only been associated with a number of cardiovascular disease processes including human post-operative atrial fibrillation [7], but is also observed to rise as early as 3 h after routine cardiopulmonary bypass [8].

The purpose of this study is therefore to determine how sVCAM-1 levels vary after CABG surgery and whether changes in sVCAM-1 follow the development of POAF. Finally, we aim to clarify whether sVCAM-1 in the pre-operative serum are predictive of POAF and its predictive ability as a biomarker of this common post-operative arrhythmia.

## Methods

### Ethics, consent and permissions

West London Regional research Ethics Committee (Ref: 09/H0711/23) approved the study. Signed informed consent was obtained from all patients prior to inclusion.

### Patient selection and recruitment

From November 2010 to September 2011, we prospectively recruited thirty-four patients undergoing non-emergent, on-pump coronary artery bypass grafting (CABG) at Imperial College Healthcare NHS Trust were to participate in this study. Exclusion criteria included emergent procedures, adjunctive procedures (e.g. valve repair or replacement), prior history of cardiac arrhythmia, thyroid disease, pre-operative anti-arrhythmic medication, and surgery utilising techniques other than standardised cardiopulmonary bypass (CPB) (e.g mini-cardiopulmonary bypass).

Pre-operatively all patients were assessed in the clinic by means of a clinical history and examination as well as electrocardiogram. Any patients with evidence of arrhythmia or history of palpitations were excluded. Continuous Holter (Novocor Vista 5 lead system, 2 channel recording) recordings were taken from all patients commencing at the time of admission until the time of surgery. Any patient displaying pre-operative arrhythmia on Holter was excluded from further inclusion in the study. Holter monitoring was subsequently continued post-operatively until the time of discharge. Atrial fibrillation was defined according to Heart Rhythm Society Guidelines [9]. Patients were categorised as developing post-operative atrial fibrillation (POAF) when there was evidence of AF of at least 30 s documented on Holter monitoring recorded after CABG without any pre-operative arrhythmic episodes [9].

### Laboratory methods

Whole blood samples were taken from all participants upon admission (24 h pre operatively) and on day 2 and day 4 post-operatively.

#### Enzyme linked immunosorbant assay

Plasma was extracted from whole blood by centrifugation at 5000 g for 6 min. Solid phase sandwich ELISA was used to quantify the concentration of VCAM1 target protein using a commercially available kit (Abcam® VCAM1 (CD106) Human ELISA Kit (ab46118)). ELISA was carried out according to the manufacturers protocol. Whole serum samples were diluted 1:50 prior to use. All samples, blanks, controls and standards were run in duplicate. 50 μl of biotynylated monoclonal antibody specific for VCAM1 was added to each well and the plate incubated for 1 h at room temperature. After washing, 100 μl of streptavidin-peroxidase enzyme was added to each well and the plate incubated again for 30 min at room temperature. After a second wash, 100 μl of chromogen TMB substrate solution was added to all wells. Plates were then incubated in the dark for 15 min at room temperature before reactions were terminated 100 μl 0.9% sulphuric acid (H_2_SO_4_). Absorbance was read immediately at a primary wavelength of 450 nm.

#### Statistical analysis

Statistical analysis was performed using student t-tests or one-way ANOVA as appropriate. Significance was considered to be met where *p* < 0.05. Evaluation of sVCAM-1 as a pre-operative biomarker for POAF was performed using predicted probability curves and diagnostic accuracy assessment using receiver operating characteristics (ROC) analysis. Calculations were performed using Graphpad Prism Version 7 (Graphpad, La Jolla, USA) and STATA version 11 (StataCorp LP, Texas, USA).

## Results

A total of 34 patients were recruited. Thirteen of these patients developed de novo atrial fibrillation in the first 5 days post-operatively. The remaining 21 patients maintained sinus rhythm throughout. Mean time to onset of post-operative atrial fibrillation was 2.5 days.

### Demographic data

Pre-operative demographics and echocardiographic parameters were comparable between the two groups. A summary of all demographic parameters is shown in Table 1. No patients suffered from pre-operative chronic infection or autoimmune disease. No patients received pre- or post- operative steroid therapy. Intra-operatively, there were no statistically significant differences in cross clamp or cardiopulmonary bypass time between the POAF and non-AF groups (X-clamp: mean non-AF 44.2 ± 13.6 vs. AF 49.4 ± 14.3 mins; *p* = 0.378. CPB time mean non-AF 77 ± 22.4 vs. AF 82.1 ± 20.7 mins; *p* = 0.596). Of the patients included, 8 underwent on-pump, beating-heart surgery without cardioplegic arrest. All other patients were performed on pump, with cardioplegic arrest and cross clamping. Only one of these developed post-operative AF and therefore no further analysis could be performed of these patients as a separate subgroup. Post-operatively, no patients suffered from significant neurological impairment. Rates of haemofiltration for significant renal dysfunction (*p* = 0.602), sternal wound infection (*p* = 0.296) and lower respiratory tract infection requiring antibiotic treatement (*p* = 0.477) were similar between POAF and non-POAF groups.

**Table 1:** Pre-operative Demographics

	AF (*n* = 13)	nAF (*n* = 21)	*p*-value
Gender (M/F)	9/4	15/6	0.716
Age (yrs)	64.6 ± 11.3	59.6 ± 12.1	0.237
BMI (kg/m^2^)	30.2 ± 6.14	27.5 ± 3.41	0.247
Height (m)	1.62 ± 0.19	1.67 ± 0.08	0.475
Weight (kg)	80.1 ± 3.86	77.7 ± 2.69	0.623
MI	33%	24%	0.555
Stroke	8.3%	14%	0.614
PVD	17%	4.8%	0.252
PCI	0.0%	0.0%	1.000
Hypertension	75%	76%	0.939
Hypercholesterolaemia	92%	90%	0.909
Family History	45%	65%	0.291
Diabetes *- On Insulin*	58% *25%*	43% *24%*	0.392 *0.939*
Smoking	17%	24%	0.629
Alcohol >10u per week	25%	25%	1.000

### VCAM-1 ELISA

**Fig. 1:**
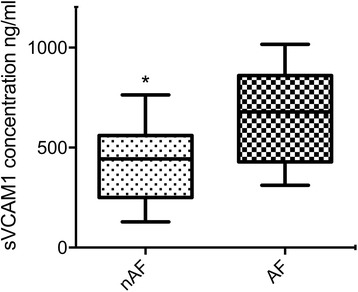
Concentration of pre-operative serum soluble VCAM-1 in AF vs. non-AF patients

Serum sVCAM-1 was significantly increased in the pre-operative serum of patients developing post-operative atrial fibrillation when compared to controls (*p* = 0.022) (Fig. 1). No significant difference was observed in sVCAM-1 levels between the POAF and non-POAF groups at 48 h and 96 h post-operatively (*p* = 0.073 and 0.135 respectively).

**Fig. 2:**
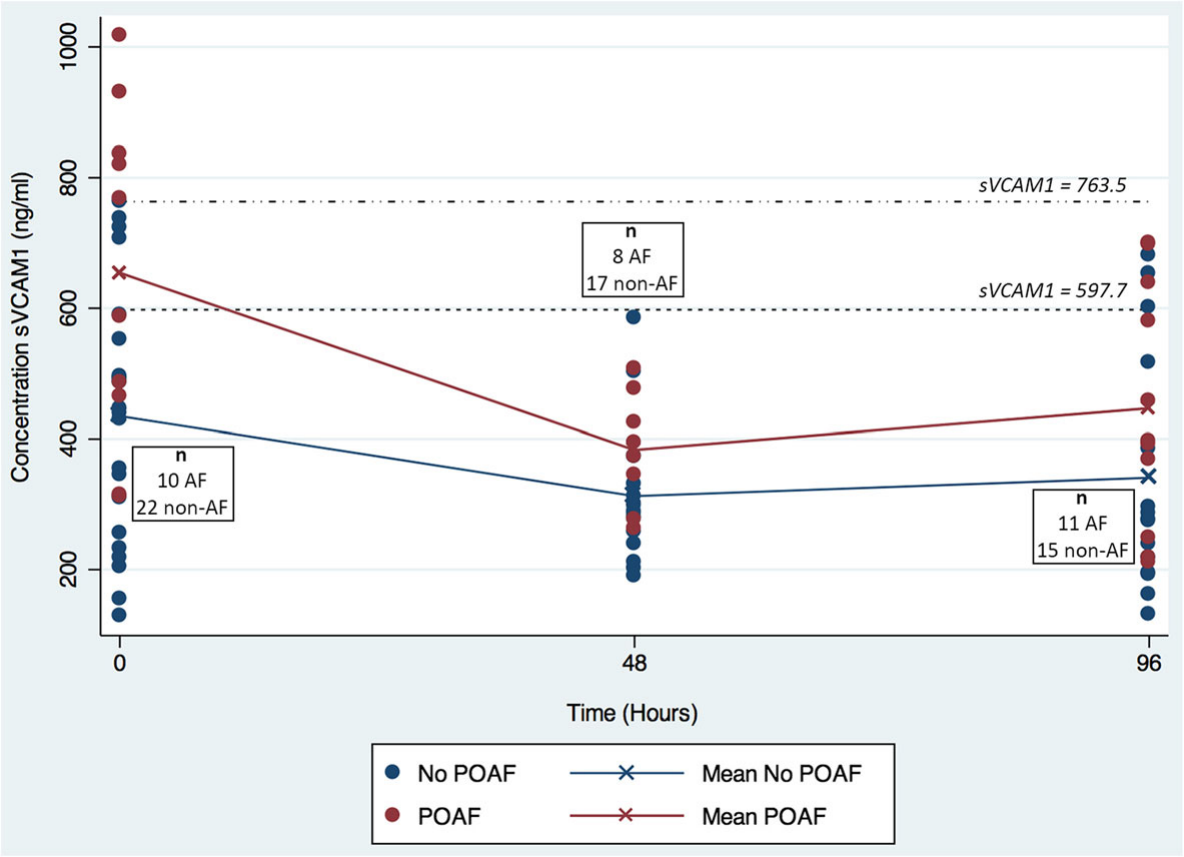
Concentration of circulating sVCAM1 in plasma samples at pre-operative, 48-h and 96-h post-operative time points

Both POAF and non-POAF groups showed an initial significant reduction in sVCAM-1 levels between pre-operative (non-AF: Mean Diff −122 ng/ml, *p* = 0.0236; POAF: Mean Diff −271 ng/ml; *p* = 0.0111) and 48-h post-operative samples (Fig. 2). Between 48-h and 96-h post operative time points there was an increase in the mean concentration of sVCAM-1 in both non-POAF and POAF groups but this was not statistically significant (non-AF: Mean Diff 28.2 ng/ml, *p* = 0.5896; POAF: Mean Diff 63.7 ng/ml; *p* = 0.3806).

No significant correlation was observed between pre-operative demographics and pre-operative serum sVCAM-1 concentration (Table 2). Similarly, there was no significant correlation between conventional markers of inflammation including blood white cell count, platelet count, C-Reactive Protein (CRP) and sVCAM-1 concentration (Table 2). The use of pre-operative statins and beta-blockers was also not found to correlate with sVCAM-1 concentrations pre-operatively (Table 2).

**Table 2:** Univariable regression of pre-operative demographics with pre-operative serum VCAM-1 concentration

	Coefficient	Standard Error	r^2	*p*-value
Gender (M/F)	38.6	88.4	0.007	0.666
Age (yrs)	3.83	3.68	0.047	0.310
BMI (kg/m^2^)	−0.16	9.56	0.000	0.987
Height (m)	215.5	350.0	0.018	0.545
Weight (kg)	3.48	3.77	0.037	0.366
MI	−26.3	90.7	0.003	0.774
PVD	−87.9	159.3	0.011	0.586
Stroke	288.3	215.5	0.062	0.192
Hypertension	61.9	90.1	0.017	0.498
Hypercholesterolaemia	154.8	130.0	0.050	0.244
Family History	−40.77	66.9	0.014	0.547
Diabetes *- On Insulin*	*113.0* *19.3*	*78.3* *90.8*	0.072 0.002	*0.160* *0.833*
Smoking	−203.4	100.1	0.133	0.052
Alcohol >10u per week	−94.8	60.76	0.086	0.131
White cell count	−22.8	40.4	0.038	0.588
Platelet count	−2.24	1.67	0.182	0.218
C-reactive protein	24.6	23.3	0.537	0.483
Beta-blocker use	−54.4	111.7	0.012	0.632
Statin use	82.8	229.8	0.007	0.723
ACE-i	70.5	104.7	0.023	0.509
Post-op medications				
Beta-blocker use	−125.9	87.19	0.077	0.161
Statin use	147.0	128.5	0.050	0.264
ACE-i	138.1	83.45	0.099	0.110

### Multinomial logistic regression

A multinomial logistic model was used to identify the impact of key clinical and univariable predictors of POAF. All univariable predictors with *p* < 0.1 were alongside the well recognised clinical predictors of age and BMI were included. Overall the model showed a good fit for prediction of POAF (p > chi2 = 0.0070, pseudo r2 = 0.5464). Pre-operative sVCAM-1 and smoking were both significant multinomial predictors of POAF (*p* = 0.026) in this model. Results are shown in Table 3.

**Table 3:** Multinomial logistic regression of key clinical predictors of AF and univariable factors with *p* < 0.10 (dependent variable: post-operative AF; independent variables: age, BMI, smoking, pre-operative serum VCAM-1) Overall model: p > chi2 = 0.0070, pseudo r2 = 0.5464

Predictor variable	Coefficient	SE	p	95% CI
Age	0.0317	0.076	0.677	−0.117, 0.181
BMI	5.468	3.060	0.074	−0.529, 11.47
Smoking	0.460	0.229	0.045	0.011, 0.909
Pre-operative sVCAM1	0.0158	0.0071	0.026	0.0019, 0.030

### Receiver operating characteristics (ROC) analysis and predicted probabilities of pre-operative sVCAM-1 as a predictor of POAF

**Fig. 3:**
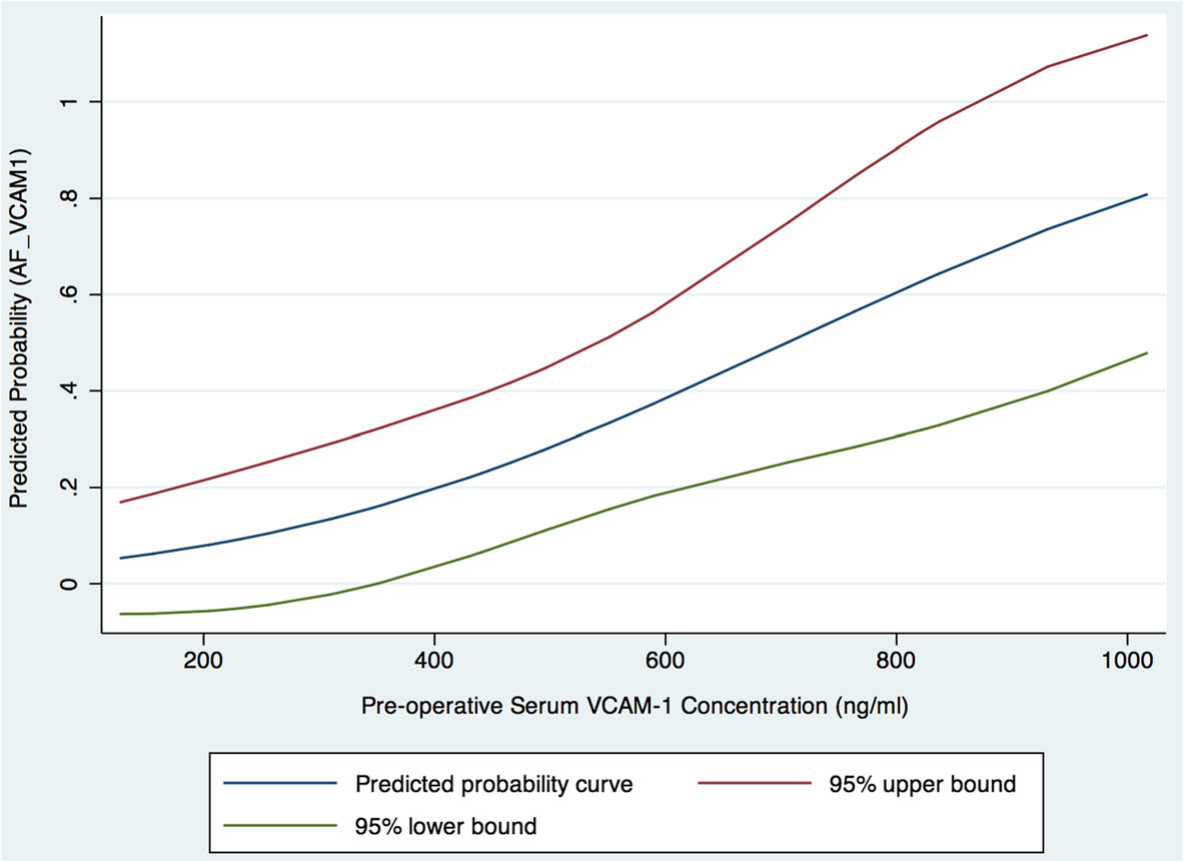
Predicted probability curve of the probability of POAF against pre-operative sVCAM-1 levels with 95% confidence intervals (Blue line- predicted probabilities; red line – 95% upper confidence limit; green line – 95% lower confidence limit)

**Fig. 4:**
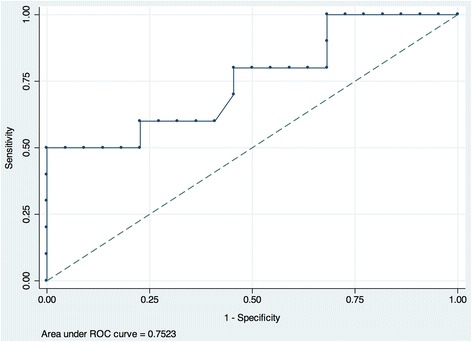
Receiver operating characteristics curve of pre-operative serum VCAM-1 as a biomarker for post-operative AF

Figure 3 demonstrates a graph of the predicted probabilities for POAF against pre-operative sVCAM-1 levels with 95% confidence intervals. The probability that patients did not develop AF given a mean sVCAM-1 concentration of 503.4 was 0.714, with a probability of POAF at this mean of 0.286. ROC analysis revealed that at a cut off of 587.7 ng/L the concentration of sVCAM-1 had a sensitivity of 60.0 and specificity of 77.27, giving a positive likelihood ratio of 2.64 and negative likelihood ratio of 0.52 for the prediction of de novo post-operative AF. The area under the curve (AUC) was 0.752 (Fig. 4).

Data were subsequently dichotomized according to this cut off value of 587.7 ng/L and outcome and predictor variables were analyzed for the separate groups. Pre-operative diabetes was the only demographic variable to be significantly different between the two sVCAM-1 cut-off groups. The number of patients with POAF did not reach statistical significance, likely given the small sample size (Table 4). A higher cut-off of 763.5 ng/L was the lowest value of sVCAM-1 allowed statistically significant prediction of POAF when patients were dichotomized into 2 groups. This corresponds to a sensitivity of 50% and a specificity of 84.38%, and accurately classified 84.4% of patients into POAF/non-POAF groups. The number of patients with POAF was significantly higher in the sVCAM-1 > 765.5 group (*p* = 0.008). No other demographic variables were statistically significant between the two groups. Results for outcome and predictor variables at this higher cut-off level are listed in Table 5.

**Table 4:** Effect of demographic variables on results as dichotomised by sVCAM-1 cut off of 597.7 ng/L

Continuous Variables	Coefficient	SE	p	95% CI
Pre-operative demographics				
Age (yrs)	0.0209	0.0383	0.585	−0.054, 0.096
BMI (kg/m^2^)	−0.0265	0.113	0.815	−0.249, 0.196
Height (m)	0.500	3.992	0.900	−7.324, 8.325
Weight (kg)	−0.004	0.0438	0.923	−0.090, 0.082
Bypass time	0.0138	0.0215	0.520	−0.028, 0.056
X-clamp time	0.0066	0.0204	0.747	−0.033, 0.047
White cell count	−0.282	0.412	0.493	−1.089, 0.525
Platelet count	−0.021	0.0225	0.356	−0.065, 0.023
Dichotomous Variables	sVCAM1 < 597.7 (*n* = 23)	sVCAM1 ≥ 597.7 (*n* = 9)	Chi^2	*p*-value
Gender (M)	76.2%	62.5%	0.5435	0.461
MI	36.4%	0%	3.5152	0.061
PVD	9.0%	0%	0.6835	0.408
Stroke/TIA	10%	14.3%	0.1545	0.694
Hypertension	63.6%	100%	3.5152	0.061
Hypercholesterolaemia	86.4%	100%	1.0647	0.302
Family History	63.6%	42.9%	0.9453	0.331
Diabetes *- On Insulin*	*40.9%* *27.2%*	*85.7%* *28.5%*	4.2693 0.0045	0.039* *0.947*
Smoking	29.4%	0%	1.9223	0.166
Alcohol >10u per week	61.9%	60%	0.7778	0.378
LMS disease	40%	50%	0.0241	0.877
Pre-operative medication				
Beta-blocker use	80%	66.7%	4.0727	0.517
Statin use	92.9%	100%	0.4511	0.502
Aspirin use	93.3%	100%	0.4200	0.517
Post-op Atrial fibrillation	22.7%	50%	2.0779	0.149

The asterisks denote standard statistical notation for significance. * - *p* < 0.05

**Table 5:** Effect of demographic variables on results as dichotomised by sVCAM-1 cut off of 763.5 ng/L (corresponds to a sensitivity of 50% and a specificity of 95.45%, with 84.38% of patients correctly classified)

Continuous Variables	Coefficient	SE	p	95% CI
Pre-operative demographics				
Age (yrs)	0.053	0.052	0.312	−0.049, 0.155
BMI (kg/m^2^)	−0.134	0.172	0.436	−0.471, 0.203
Height (m)	−0.229	4.151	0.956	−8.36, 7.91
Weight (kg)	−0.047	0.050	0.351	−0.146, 0.052
Bypass time	0.010	0.026	0.708	−0.041, 0.061
X-clamp time	−0.011	0.025	0.673	−0.060, 0.038
White cell count	−0.301	0.488	0.537	−1.257, 0.655
Platelet count	−0.029	0.031	0.352	−0.090, 0.032
Dichotomous Variables	sVCAM1 < 763.5 (*n* = 26)	sVCAM1 ≥ 763.5 (*n* = 6)	Chi^2	*p*-value
Gender (M)	75%	60%	0.4661	0.495
MI	32%	0%	1.7676	0.184
PVD	8%	0%	0.3437	0.558
Stroke/TIA	8%	25%	1.0745	0.300
Hypertension	68%	100%	1.768	0.184
Hypercholesterolaemia	88%	100%	0.5354	0.464
Family History	64%	25%	2.1622	0.141
Diabetes *- On Insulin*	*48%* *24%*	*75%* *50%*	1.0067 1.1669	*0.316* *0.280*
Smoking	20%	0%	0.9667	0.326
Alcohol >10u per week	62.5%	25%	1.9688	0.161
LMS	31.8%	25%	0.0739	0.786
Pre-operative medications				
Beta-blocker use	73.9%	50%	0.9345	0.334
Statin use	100%	87.0%	0.5870	0.444
Aspirin use	100%	91.3%	0.3757	0.540
Post-op Atrial fibrillation	20%	75%	7.143	0.008**

The asterisks denote standard statistical notation for significance. ** - *p* < 0.005

## Discussion

These results have identified significantly higher levels of sVCAM-1 in the pre-operative serum of POAF patients, supporting the hypothesis that POAF is associated with pre-existing endothelial activation and systemic inflammation. Furthermore, these findings suggest that sVCAM-1 may act as a prospective biomarker able to predict POAF prior to coronary artery bypass surgery.

Vascular cell adhesion molecule 1 (VCAM-1) is a member of the immunoglobulin superfamily responsible for the mediation of signal transduction, cell adhesion and transendothelial migration of dendritic and macrophage-like cells. As such, it plays a key role in mediating the cellular response to inflammation [6]. Soluble VCAM-1 (sVCAM-1) is released from endothelial cells and leukocytes in response to cytokine activation. As sVCAM-1 is predominantly a marker of endothelial cell activation in vivo, it has previously been studied both in determining the inflammatory response to cardiopulmonary bypass and in the development of POAF. In their 2002 study, Andresen et al. demonstrated that sVCAM-1 levels peaked at 8 h following CPB, suggesting early endothelial cell activation in response to contact of human blood with the foreign material of the bypass circuit [8].

Inflammation is thought to play a key role in the pathogenesis of de novo post-operative atrial fibrillation. Indeed, a key clinical observation is the association of POAF with post-operative infection, whereby sepsis mediated inflammation and oxidative stress is thought to stimulate a cytokine surge that subsequently leads to both electrical and structural changes that promote increased automaticity and autonomic dysfunction leading to an increased risk of atrial fibrillation [10].

The role of sVCAM-1 in POAF was first identified by Verdejo et al. in their 2011 study that demonstrated increased levels of circulating sVCAM-1 in patients with no prior history of arrhythmia who went on to develop AF post-operatively [7]. Furthermore, pre-operative sVCAM-1 appeared to be a more reliable predictor of POAF than other markers of systemic inflammation such as hsCRP and IL-6, which are often confounded by age and other medical co-morbidities [11]. As sVCAM-1 more specifically reflects endothelial cell activation, it has been suggested that endothelial dysfunction and thus susceptibility to CPB related ROS generation, may be a key factor underlying the pathogenesis of POAF [7].

This study confirms the findings of Verdejo and colleagues’ [7] demonstrating significantly higher levels of sVCAM-1 in the pre-operative serum of POAF patients compared to those maintaining sinus rhythm after CABG surgery. Moreover, ROC analysis revealed that at a fold change cut off of 587.7 ng/ml sVCAM1 was associated with a diagnostic accuracy of 75.2% in the prediction of de novo POAF in our patients.

Beyond the measurement of pre-operative sVCAM-1, our results have also examined serum sVCAM-1 expression at 2 time points corresponding to the time to peak onset of POAF. At 48 and 96 h post-operatively, sVCAM-1 concentration remained higher in the POAF than non-POAF group; however, these differences were no longer significant. Interestingly, in both groups sVCAM-1 concentration fell significantly between pre-operative and 48 h time points, with a greater reduction in mean sVCAM-1 concentration in the POAF group (Mean Diff −271 vs. -122 ng/ml). This result initially seemed counter-intuitive, as we had expected an increase in sVCAM-1 levels post-operatively in line with the overall inflammatory response to cardiopulmonary bypass. However, our findings follow similar observations by Doo et al. who in their study of 55 consecutive patients undergoing coronary stenting did not observe any increase in sVCAM-1 either at 24 or 72 h post intervention [12]. As previously noted, serum VCAM-1 levels have been shown to peak at 8 h post CPB [8] and it is therefore likely that the transient increase in sVCAM-1 in the immediate post-operative/post-procedural period, is no longer apparent when measured at 24–48 h. However, the reason for a fall in sVCAM-1 below baseline concentration still remains unclear. It is possible that these observations may reflect the release of other pro-inflammatory cytokines in the post-operative or post-procedural period, which act to suppress sVCAM-1. One potential candidate for this is IL-8, a pro-inflammatory cytokine that stimulates neutrophil chemotaxis and is known to be significantly increased in the systemic circulation after cardiopulmonary bypass, remaining elevated at 48 h post-operatively [13]. In the setting of altered vascular sheer stress, similar to the disruption of normal flow dynamics known to occur as a result of cardiopulmonary bypass, IL-8 may also act to reduce the activation of p38 and NF-κB thus blocking smooth muscle release of VCAM-1 and reducing circulating sVCAM-1 concentrations [14]. As such, increased IL-8 following CPB may offer a potential explanation for the post-operative reduction in sVCAM-1 in this patient cohort.

### Limitations

The results of this study should be considered in the context of their limitations. Firstly, this is a small study, and with this in mind we cannot exclude the possibility of type II error particularly when considering sVCAM-1 levels at 48 and 96 h timepoints where sample size is particularly limited. As such, these results should be considered a platform on which to build future work and should be validated prospectively in a much larger patient cohort. Secondly, we have not attempted to determine any causal link between VCAM-1 and the development of POAF, and as such our findings do not offer mechanistic information regarding the pathophysiology of POAF. Indeed, it is likely that as with biomarkers such as pro-BNP and C-reactive protein, VCAM-1 represents a measurement of an underlying pro-inflammatory state, the cause of which may itself be multifactorial. Furthermore, we cannot exclude the possibility that sVCAM-1 is a marker of another, more sensitive, clinical attribute and that it is this attribute that predicts POAF rather than sVCAM-1 which may be itself a bystander. Third, a number of confounding factors may also influence the presence or absence of pre-operative inflammation. Although no significant differences were observed in pre-operative co-morbidities, echocardiographic parameters or medical therapy, the presence of sub-clinical sepsis either pre- or post-operatively cannot be excluded. In particular, it is notable that when examining the dichotomized data only pre-operative diabetes was statistically different between sVCAM-1 groups below and above the optimal cut-off for biomarker development (587.7 ng/L) suggested by ROC analysis. Both type 1 and type 2 diabetes have been associated with increased levels of sVCAM-1 in previous studies, particularly in the setting of renal dysfunction and hypertension [15, 16]. As such, at this lower cut-off value the presence of diabetes may significantly confound the predictive role of sVCAM-1 as a pre-operative biomarker of POAF. At a higher cut-off value of 763.5 ng/L signifying higher specificity for AF (84.8%) only POAF was significantly different between high- and low- sVCAM groups. These results suggest that a higher cut-off value may be necessary to develop a pre-operative biomarker sufficiently specific for POAF and further research is required in much larger patient cohorts to examine in more detail the impact of confounders such as diabetes.

## Conclusions

Evaluation of serum VCAM-1 in this patient cohort corroborated previous findings that patients developing POAF express higher levels of pre-operative sVCAM-1 than those maintaining sinus rhythm, and in addition showed a significant fall in sVCAM-1 expression within 48 h post-operatively. These findings together support the hypothesis of a systemic inflammatory substrate for this arrhythmia and highlight the potential for biomarker development.

## Abbreviations

AF: Atrial fibrillation
CABG: Coronary artery bypass grafting
CPB: Cardiopulmonary bypass
ELISA: Enzyme linked immunosorbant assay
IL: Interleukin
POAF: Post-operative atrial fibrillation
ROC: Receiver operating characteristics
ROS: Reactive oxygen species
TNF: Tumor necrosis factor
VCAM-1: Soluble vascular endothelial cell adhesion molecule
